# In vivo aging-induced surface roughness alterations of Invisalign^®^ and 3D-printed aligners

**DOI:** 10.1177/14653125221145948

**Published:** 2022-12-27

**Authors:** Despina Koletsi, Nearchos Panayi, Christodoulos Laspos, Athanasios E Athanasiou, Spiros Zinelis, Theodore Eliades

**Affiliations:** 1 Clinic of Orthodontics and Pediatric Dentistry, Center of Dental Medicine, University of Zurich, Zurich, Switzerland; 2 Department of Dentistry, School of Medicine, European University Cyprus, Nicosia, Cyprus; 3 Private practice, Limassol, Cyprus; 4 Department of Biomaterials, School of Dentistry, National and Kapodistrian University of Athens, Athens, Greece

**Keywords:** 3D-printed, aligners, Invisalign^®^, optical profilometry, roughness, surface characterisation

## Abstract

**Objective:**

To assess the surface roughness of in-house 3D-printed orthodontic aligners compared with Invisalign^®^ appliances, both retrieved as well as in the ‘as-received’ control status.

**Design:**

An in vitro study following intra-oral material aging.

**Setting and participants:**

Twelve clinically used Invisalign^®^ appliances and the same number of 3D-printed aligners, without involvement of attachments, were obtained from a respective number of patients. A similar number of ‘as-received’ aligners, of each material, were used as control (CON) groups.

**Method:**

Four groups of materials were examined: A = Invisalign^®^ CON; B = Invisalign^®^ used; C = 3D-printed CON; and D = 3D-printed used. Optical profilometry was employed to examine the following surface roughness parameters: amplitude parameters Sa, Sq and Sz and functional parameters Sc and Sv. Descriptive statistics and quantile regression modeling were conducted, and the level of statistical significance was set at α = 0.05.

**Results:**

Intra-oral exposure of 3D-printed aligners was significantly associated with increase in all tested parameters (*P* < 0.001 at all occasions). Significant differences were detected in the retrieved 3D-printed aligners compared with Invisalign^®^ retrieved, with the exception of Sz. The respective effect sizes (median differences) were as follows: Sa: 169 nm, 95% confidence interval [CI] = 89–248, *P* < 0.001; Sq: 315 nm, 95% CI = 152–477, *P* < 0.001; Sc: 233 nm^3^/nm^2^, 95% CI = 131–335, *P* < 0.001; and Sv: 43 nm^3^/nm^2^, 95% CI = 17–68, *P* = 0.002.

**Conclusion:**

Within the limitations of this study, we concluded that surface roughness differences existed between 3D-printed aligners and Invisalign^®^ in the retrieved status, as well as between the control and retrieved 3D-printed groups.

## Introduction

Aesthetic demand and patients’ need have been dictating orthodontic treatment decisions and preferences for years following both clinicians’ and patients’ views. It has been reported that seeking ‘invisible’ orthodontic modalities, especially by adult patients, has attained an increasing interest over the recent years, as also documented by clinicians’ files and records worldwide (d’Apuzzo et al., 2019; Hennessy and Al-Awadhi, 2016). The use of orthodontic aligners as a primary representative of ‘invisible’ orthodontics has gained much attention from researchers, in an attempt to frame the basis over their efficacy to achieve the desirable treatment outcome compared to the ‘gold standard’ of fixed orthodontic appliances (Koletsi et al., 2021; Papageorgiou et al., 2020). In addition, oral hygiene, safety considerations and further material properties characterisation parameters of aligners have been studied under the prism of potential risk identification for the patients (Eliades and Koletsi, 2020; Iliadi et al., 2020; Oikonomou et al., 2021; Papadopoulou et al., 2019; Suter et al., 2020).

To date, the aforementioned research perspectives have been designed and applied mostly to commercially available clear aligners or thermoplastic materials used for their fabrication. Lately, immense interest been documented related to the adoption of ‘in- house’ 3D-printed technology for medical applications, while dentistry, orthodontics and ‘in- office’ associated workflow, have not been left aside (Edelmann et al., 2020; Panayi and Eliades, 2022). The concurrent ‘do-it-yourself’ hype has gradually allowed the formation of a currently unstable and ground-breaking evidence base in the field of 3D printing in orthodontics, related to in-house aligner fabrication (Panayi and Eliades, 2022; Zinelis et al., 2022). Apparent advantages, stemming from the cost-effectiveness or the simplicity and swiftness characterising their fabrication, need to be critically examined within the frame of potential gains or hazards for the end user (both operator and patient). Clinical effectiveness, mechanical properties, aging and safety concerns formulate the basis for continuing and novel research endeavors in the field (Can et al., 2022; Edelmann et al., 2020; Pratsinis et al., 2022).

Empirical data from a recent report on the effect of in vivo aging on the mechanical properties of a 3D-printed aligner material, has revealed that intra-oral service of the aligners for a period of one week, does not lead to significant alterations in hardness, indentation modulus, elastic index and indentation relaxation (Can et al., 2022). Interestingly, on a subsequent spectroscopic analysis, the chemical composition, of such an in-house fabricated material, has revealed significant differences from the commercially available thermoformed orthodontic aligners (Can et al., 2022). However, evidence has shown that the mechanical properties may be largely dependent on the 3D printer used for fabrication (Zinelis et al., 2022). Furthermore, the digital design and 3D printing of orthodontic aligners have been documented to result in products with increased thickness, thus, questioning the effectiveness of the clinical utility of the appliances (Edelmann et al., 2020). In terms of the biological profile of the aligners, a very recent publication has revealed no evidence of cytotoxic or estrogenic activity induced by a 3D-printed aligner eluent sample during a two-week aging (Pratsinis et al., 2022). Currently, there is lack of information on the surface characterisation of 3D ‘in-office’ fabricated orthodontic aligners; in this respect, data from commercial Invisalign^®^ appliances in-service demonstrate that surface roughness is invariably affected after one week of intra-oral use, potentially contributing to biofilm adhesion and plaque accumulation (Papadopoulou et al., 2019).

Thus, the aim of the present study was the characterisation of surface profile of in- house 3D-printed orthodontic aligners compared to Invisalign^®^ appliances (Align Technology, San Jose, CA, USA) both retrieved after intra-oral service as well as in the ‘as-received’ control status. The null hypothesis was that no significant differences exist between the materials and, following their clinical use, regarding roughness parameters.

## Materials and methods

### Sample collection process

Twelve clinically used Invisalign^®^ (Align Technology, San Jose, CA, USA) appliances (material: SmartTrack™) and the same number of 3D-printed aligners were obtained from patients with no history of parafunctional habits. The aligners were retrieved from two dental practices (NP, CL) following their use in consecutively treated patients who fulfilled the inclusion criteria. Each practice exclusively used a protocol of either Invisalign^®^ or 3D-printed aligners. All patients and/or their legal guardian provided informed consent and the relevant national Dental Association approved the study design, which dealt with anonymised collection of retrieved materials, placed during routine treatment of patients and, which, after the term of their service would have been otherwise discarded (Reference No. 250311NP). A respective number of ‘as-received’/not-used aligners (both Invisalign and 3D-printed, 12 each) were used as control (CON) groups. Thus, four groups of materials were examined: A = Invisalign^®^ CON; B = Invisalign^®^ used; C = 3D-printed CON; and D = 3D-printed used. Treatment with aligners included no attachment adjuncts and overall a similar degree of arch variability (mainly anterior crowding). The wearing protocol for the Invisalign^®^ was seven days and this was also followed for the 3D-printed counterparts, also based on relevant evidence on the topic, regarding considerable force decay within the first week of utilisation (Iliadi et al., 2021). All patients followed the same aligner cleaning regimes and wear/care instructions. They were instructed to wear the aligner >20 h per day and brush the aligner once a day. Compliance was checked through a questionnaire addressing compliance.

The 3D-printed aligners were fabricated using the Moonray S100 printer (Sprintray, Los Angeles, CA, USA). The tank was filled with the TC-85DAC resin (Graphy, Seoul, Korea) indicated for manufacturing of clear aligners. The aligners were printed in successive layers of 100-μm nominal size in 70 min, employing a 405-nm blue-violet light and digital light processing technology. A centrifugation machine was used to remove excess resin for 4 min. Then the aligners were post-cured for 12 min from the cervical and incisal sides in a post-curing unit (Cure M; Graphy, Seoul, Korea).

The specimens underwent thorough and non-abrasive chemical cleaning (Steraligner, TJA Health, LLC, Joliet, IL, USA) before assessment, thus removing the accumulated plaque and calculus remnants.

### Optical profilometry

A small area (approximately 4 × 4 mm) was cut with a lancet from the lingual surface of the central incisor region and the internal part (that was in contact with tooth) was analysed using optical profilometry. An optical profilometer (Wyko NT-1100,Tuscon, AZ, USA) with a 20× nominal magnification lens was employed accordingly, and the acquisition area involved a rectangle with dimensions of 231 × 303 μm. In order to remove the effect of waviness, a Gaussian regression filter was applied with a 0.025-mm short wavelength cutoff filter and then the following surface roughness parameters were determined: arithmetic mean deviation (Sa); root mean square root (Sq); maximum height of the surface (Sz); core void volume showing the volume of the surface (Sc) that could support 10%–80% of the bearing ratio; and the surface volume (Sv) showing the volume of the surface that could support 80%–100% of the bearing ratio. The bearing ratio curve was plotted by passing a horizontal level downward through the surface, from the highest peak to the deepest valley. All acquired data were analysed using Vision 64, version 5.7 software (Bruker Corporation, Tucson, AZ, USA).

### Statistical analysis

Data were checked for normality of residuals for all surface roughness parameters statistically through the Shapiro–Wilk test and visually through q-q plots and q-norm plots (quantiles of a variable against quantiles of normal distribution). Descriptive statistics included median values and interquartile range (IQR), following the non-normal distribution of the residuals. A median (quantile) regression analysis was built (‘qreg’ command) with material (aligner) type and intra-oral service (retrieved or non-used) as predictors. The interaction between predictors was tested through a likelihood ratio test. In the presence of interaction, stratum specific estimates are presented to facilitate straightforward interpretation of the data. The level of statistical significance was set at α = 0.05. All statistical analyses were performed with Stata version 15.1 (Stata Corp, College Station, TX, USA).

## Results

**Figure 1.**
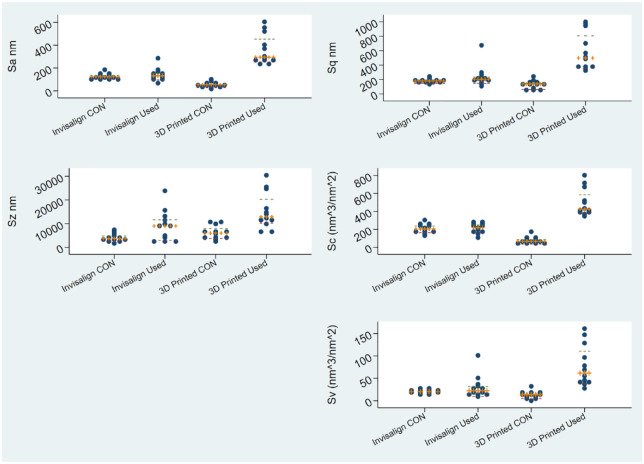
Dot plots on all tested groups of aligners, across roughness parameters. Please note the differences on the y-axis scale.

**Figure 2.**
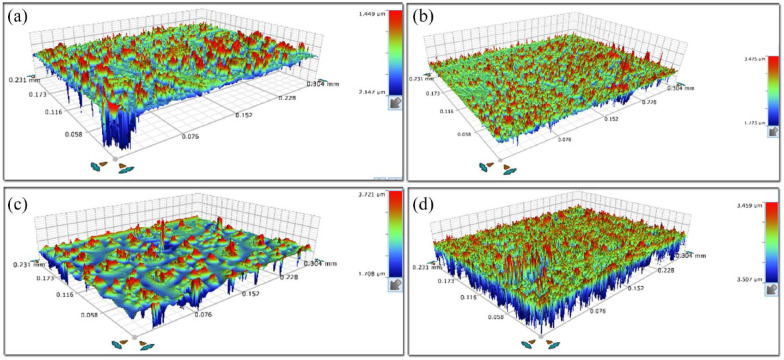
Representative 3D optical profilometric images from all groups tested. (a) Invisalign control, (b) Invisalign retrieved, (c) 3D-printed control and (d) 3D-printed retrieved. Please note the differences between scale.

The results of the present study are given both descriptively (Table 1, Figure 1) and through regression modeling (Table 2). Non-normal distribution of the residuals was confirmed. Figure 2 demonstrates representative 3D reconstruction images of all groups tested and the parameters’ variability, conditional on type of material/ aligner and effect of intra-oral service, is depicted. A significant interaction effect was identified for all parameters tested but one (Sz), between material (Invisalign^®^/3D-printed aligners) and intra-oral service (yes/no). There was strong evidence that intra-oral service of 3D-printed aligners was associated with an increase in all tested parameters (*P* < 0.001) (Table 2). In addition, significant differences were detected in retrieved 3D-printed aligners compared to Invisalign^®^ retrieved counterparts in all but one parameter, demonstrating solid evidence of considerably higher amount of roughness parameters’ values in the 3D-printed group of specimens. For example, regarding the effect on Sa in the stratum-specific estimates (Table 2), the 3D-printed aligners presented a median of 169-nm higher values than Invisalign^®^ when retrieved (β coefficient, median difference = 169 nm; 95% confidence interval [CI] = 89–248; *P* < 0.001). Likewise for the parameters Sq (β coefficient, median difference = 315 nm; 95% CI = 152–477; *P* < 0.001), Sc (β coefficient, median difference = 233 nm^3^/nm^2^; 95% CI = 131–335; *P* < 0.001) and Sv (β coefficient, median difference = 43 nm^3^/nm^2^; 95% CI = 17–68; *P* = 0.002). To be specific, only in the Sz parameter was there an absence of evidence of a considerably higher value in the 3D-printed group compared to the Invisalign group, after adjusting for intra-oral service, with a relatively wide range of the confidence intervals (adjusted β coefficient, median difference = 2739 nm; 95% CI = −1043 to 6520; *P* = 0.15).

**Table 1.** Descriptive statistics on roughness parameters for all tested groups (n = 12 in all groups).

Roughness parameter	Invisalign CON	Invisalign used	3D-printed CON	3D-printed used
	Median (IQR)			
Sa (nm)	124 (1067–143)	131 (104–164)	54 (36–66)	295 (264–464)
Sq (nm)	182 (161–200)	203 (166–247)	138 (77–162)	499 (373–822)
Sz (nm)	3671 (2981–5260)	9002 (3452–12,153)	5995 (4192–8414)	12,842 (10,656–20,836)
Sc (nm^3^/nm^2^)	204 (174–247)	219 (168–261)	66 (49–96)	425 (394–597)
Sv (nm^3^/nm^2^)	20 (17–22)	22 (12–34)	13 (7–18)	62 (40–113)

IQR, interquartile range.

**Table 2.** Quantile regression for the examined roughness parameters (n = 48).

Predictor variables	β-coefficient	95% CI	*P* value
	Sa (nm)*		
CON (non-used)			
Invisalign	Reference		
3D-printed aligners	−67	−147 to 12	0.10
Used			
Invisalign	Reference		
3D-printed aligners	169	89–248	<0.001
Invisalign			
CON	Reference		
Used	12	−68 to 91	0.77
3D-printed			
CON	Reference		
Used	248	168–327	<0.001
	Sq (nm)*		
CON (non-used)			
Invisalign	Reference		
3D-printed aligners	−46	−208 to 117	0.57
Used			
Invisalign	Reference		
3D-printed aligners	315	152–477	<0.001
Invisalign			
CON	Reference		
Used	25	−138 to 187	0.76
3D-printed			
CON	Reference		
Used	385	223–548	<0.001
	Sz (nm)^†^		
Material			
Invisalign	Reference		
3D-printed aligners	2739	−1043 to 6520	0.15
Intra-oral service (used)			
No	Reference		
Yes	5660	1879–9441	0.004
	Sc (nm^3^/nm^2^)*		
CON (non-used)			
Invisalign	Reference		
3D-printed aligners	−149	−251 to −47	0.005
	Sc (nm^3^/nm^2^)*		
Used			
Invisalign	Reference		
3D-printed aligners	233	131–335	<0.001
Invisalign			
CON	Reference		
Used	−9	−110 to 93	0.87
3D-printed			
CON	Reference		
Used	373	271–475	<0.001
	Sv (nm^3^/nm^2^)*		
CON (non-used)			
Invisalign	Reference		
3D-printed aligners	−6	−32 to 19	0.63
Used			
Invisalign	Reference		
3D-printed aligners	43	17–68	0.002
Invisalign			
CON	Reference		
Used	7	−19 to 32	0.59
3D-printed			
CON	Reference		
Used	56	30–81	<0.001

Predictor variables are material and intra-oral service of the appliance. Please note that in the models that include interaction terms, stratum specific estimates are presented (following likelihood ratio test confirmation for significant interaction).

* Interaction for material*use significant *P* < 0.001.

† No interaction detected for Sz (*P* = 0.15).

CI, confidence interval; Sa, arithmetic mean deviation; Sc, core void volume; Sq, root mean square root; Sv, surface volume; Sz, maximum height of the surface.

## Discussion

The present study provides first empirical data on surface characterisation of 3D-printed aligners, fabricated ‘in-house’. It gives further insights regarding the effect of intra-oral service of the aligners and compares surface characteristics of these appliances to a widely used manufactured product. The confirmation of strong evidence of substantial differences related to the material under examination, as well as the effect of intra-oral service, has led to the rejection of the null hypothesis. Overall considerable and significant differences in all roughness parameters were identified for the retrieved 3D-printed aligners compared to a similarly constructed control group, but also compared to retrieved Invisalign^®^ aligners. The detected rise in roughness parameters after the tested 3D-printed aligners were ‘in-service’ is suggestive of inbred characteristics and material integrity of the appliances being exposed to real-life clinical situations and intra-oral conditions.

A previous report examined roughness and surface properties of commercially available aligners in-service for up to two weeks (Papadopoulou et al., 2019), with no further exploration of any types of ‘in-office’ printed material. Interestingly, the magnitude and values of a number of roughness parameters tested were significantly reduced after intra-oral aging of one week, but remained stable thereafter. This is in contrast with the findings of the present study, at least for the commercially used appliances (Invisalign^®^); however, the substantial differences in the settings of those studies should not be neglected. The study of Papadopoulou et al. (2019) used Invisalign^®^ appliances coupled with attachment use and the respective testing areas included both the internal surface of the aligners attachments as well as the adjacent lingual surface in contact with the aligner. This might have induced the effect of wear, resulting in subsequently smoother surfaces following intra-oral service and function and multiple rounds of removal and re-fitting of the aligner, thus acting in a polishing-like manner. In the present study, included aligners were free of attachments when in-service.

It has been documented that to achieve better understanding of the surface morphology of a tested material, a number of parameters is deemed necessary to fully frame its characterisation (Stout and Blunt, 2000). As such, we examined both amplitude (Sa, Sq, Sz) as well as functional parameters (Sc, Sv). The former parameters inform about the increased amplitude heights (peak and valley slopes) and resulted in increased material surface in the retrieved ‘in-office’ fabricated aligners. The latter are indicative of the core surface characterisation of the material and have been related to the fluid retention capacity of the material: the Sc after excluding the shallowest 10% and the deepest 20% and the Sv focusing on the largest 20% of the valleys at the bearing ratio. Considering the significant increase in the functional parameters after intra-oral exposure detected solely for the printed aligners group, one might presume that these appliances might be more prone to plaque accumulation and plaque retention, thus raising concerns for their clinical safety and potential adverse effects on the maintenance of oral hygiene and relative impact throughout the treatment course (Dutra et al., 2018; Levrini et al., 2015). In addition, the combined effect and simultaneous action of multiple intra-oral vectors and conditions during aligner treatment, such as impediments of constant saliva cleansing of the tooth surface, tongue activity, buccal soft tissue cleansing and calculus formation, may potentially also lead to aligner discoloration or pigmentation (Liu et al., 2016). Albeit these reasonable speculations on novel 3D-printed aligner material following close examination of surface microtopography, a recent systematic review and meta-analysis on oral hygiene parameters revealed that orthodontic treatment with the commercially available Invisalign^®^ appliances, without the use of any attachments or adjuncts, was associated with lower plaque scores than the respective ‘gold standard’ of fixed appliances (Oikonomou et al., 2021). The Invisalign^®^ appliances in the present study did not show evidence of surface characterisation deterioration after intra-oral exposure and service. In essence, for the Invisalign^®^ group of retrieved aligners, the sole parameter that demonstrated a clear trend and dynamic to raise after use was the amplitude parameter Sz; however, in contrast to the rest of the parameters, a substantial variability was detected reaching the levels of approximately 9000 nm in the IQR range for a very similar median value. Furthermore, consideration of Sz in isolation may not be informative of the aggregate surface topography of a tested material since it describes the maximum peak and valley height, representing the gross irregularity status of the material surface (Eliades et al., 2004), or it might also be subject to measurement noise, high variability or contamination due to the dependence on maximum values (Sacerdotti et al, 1999, 2000).

Findings related to the mechanical property profile of manufactured aligners, either long-established or recently appearing 3D-printed counterparts, have also not been unanimous in the literature (Bradley et al., 2016; Can et al., 2022; Papadopoulou et al., 2019), thus revealing gaps in the knowledge perspective regarding their clinical effectiveness and a predictable clinical outcome (Koletsi et al., 2021). It has been shown that bulk properties of Invisalign^®^ appliances, after intra-oral aging approaching 4–8 weeks, may present detrimental alterations. Interestingly, after exposure in the oral environment and function, the aligners showed increased deformation, a more brittle behavior and a decreased wear resistance (Bradley et al., 2016). Such effects were also confirmed by a recent report on one- to two-week aged Invisalign^®^ material coupled with the use of attachments (Papadopoulou et al., 2019). In contrast, evidence from a first report on ‘in-office’ printed aligners, fabricated in a similar manner to the present study, has demonstrated a sound retention potential of the mechanical profile of the appliances, one week after intra-oral service, with minimal and non-significant or clinically relevant differences being revealed in hardness, indentation modulus, elastic index and relaxation index between the appliances before and after use (Can et al., 2022). However, it has been reported that stress relaxation of such ‘do-it-yourself’ appliances is almost 10 times more compared to Invisalign^®^ (Jaggy et al., 2020; Papadopoulou et al., 2019). This might bear implications for the clinical use of the appliances and material integrity, potentially resulting in a softer end-product for use, which might in turn provide some critical background information for the identified surface microtopography alterations demonstrated after intra-oral exposure of 3D-printed aligners in the present study. Consequently, any wear associated with the degradation potential of the polyester-urethane-polymer nature of the printed aligners coupled with its softer matter, when in-service and subject to the continuous trigger within the intra-oral environment, might result in particulate production and leaching of compounds and substances in the oral cavity (Iliadi et al., 2020). The hazards associated with the latter are currently unknown, although recent evidence on in vitro levels of cytotoxicity and estrogenicity of these materials is not discouraging (Pratsinis et al., 2022); however, the effect on in vivo aging and intra-oral function are yet to be established.

Nevertheless, the findings of this report should be interpreted with caution and a more secure evidence base should be provided through further research in the field before any attempt to generalise the results. We studied for the first time the surface material properties of ‘in-office’ aligners fabricated by a single printer, using a single resin matter. It should be noted that the uniformity of material properties of printed aligners fabricated through different 3D-printers has been questioned, at least on what affects mechanical properties (Zinelis et al., 2022); this might also bear an impact on the surface characterisation of the materials. Certainly, variabilities in the manufacturing technology, the printing process and apposition of resin layers; the resin layer depth, the depth of cure, the strength of interlayer bonding between successive printed layers, the post-curing process, the oxygen-inhibited layer effect, condition, time and direction are risk factors that may compromise consistency and accuracy of the fabrication process and consequently result in end-products of non-similar or non-comparable surface properties (Al-Dulaijan et al., 2022; Stansbury and Idacavage, 2016).

## Conclusion

Acknowledging all caveats and applicability perspectives, the findings of the present study constitute the first empirical evidence on the surface characterisation and in vivo aging of 3D- printed aligners and allowed comparisons with long-standing company manufactured counterparts. Evidence revealed that intra-oral exposure and function induced significant and substantial changes in surface roughness properties of ‘in-house’ fabricated aligners at all levels. The interconnection with clinical efficiency and safety, in the terms of a course of orthodontic treatment with such appliances, remains to be established, if present. Currently, there is no evidence base in this respect and further clinical research is deemed imperative.
